# Invasive Breast Carcinoma: Rare Clinical Presentation in a Male Patient

**DOI:** 10.7759/cureus.20547

**Published:** 2021-12-20

**Authors:** Mariam Hanna, Miranda Solly

**Affiliations:** 1 Radiology, University of Florida College of Medicine, Gainesville, USA

**Keywords:** axillary lymphadenopathy, lymphadenopathy, male breast tumor, brca gene mutation, invasive ductal cell carcinoma

## Abstract

A 74-year-old male presented for evaluation of a right breast mass that the patient had self-detected a few weeks prior. This gentleman had an extensive family history of cancer, with a father (70 years old) and daughter (31 years old) with breast cancer, a sister with rectal cancer (70 years old), and a son and daughter with a pathogenic variant of the breast cancer (BRCA) gene. As with women, the frequency of malignancy increases with age. Many similar risk factors are noted to overlap between male and female presentations. Men tend to have similar profiles and presentations as postmenopausal women. The vast majority are diagnosed early, and less than 5% present with metastatic disease. This case report highlights the imaging presentation of this rare diagnosis and reviews its etiology and pathophysiology.

## Introduction

Male breast cancer is exceedingly rare, accounting for less than 1% of all cancers in men and less than 1% of all breast cancer diagnoses worldwide [1,2]. Male breast cancer is very similar in terms of epidemiology to postmenopausal female breast cancer, with similar risk factors including age, family history, pathogenic mutations in the breast cancer (BRCA) genes, external use of estrogen and testosterone, and obesity, among others [1,2]. A pathogenic mutation in the BRCA2 gene is the most relevant risk factor currently described for the development of male breast cancer [3]. Breast cancer affects 5-10% of men with BRCA2 mutations over their lifetime [4,5]. The National Comprehensive Cancer Network (NCCN) guidelines recommend that men with BRCA mutations undergo a yearly clinical breast examination starting at the age of 35 years [6]. The imaging guidelines for men are limited and do not include strong recommendations [6]. Most cases of breast cancer in men are invasive carcinomas, with invasive ductal carcinoma as the most common type. Less common histologic subtypes in men include papillary cancers and mucinous cancers, with the least common being lobular carcinoma [6]. Cancers in men are more likely to be positive for estrogen receptors and negative for human epidermal growth factor receptor 2 (HER2). The following is a case discussion and presentation of a male patient with breast cancer. 

## Case presentation

Physical examination

**Figure 1:**
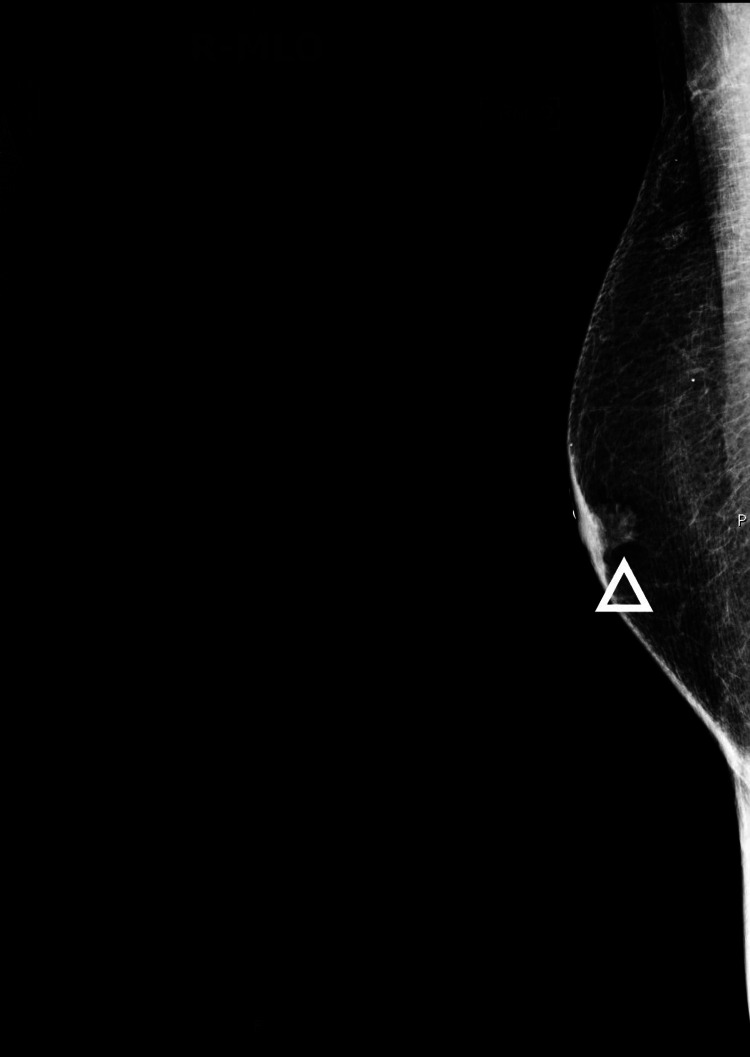
Right breast mediolateral oblique view diagnostic mammogram shows a high-density mass with spiculated margins in the right subareolar region.

**Figure 2:**
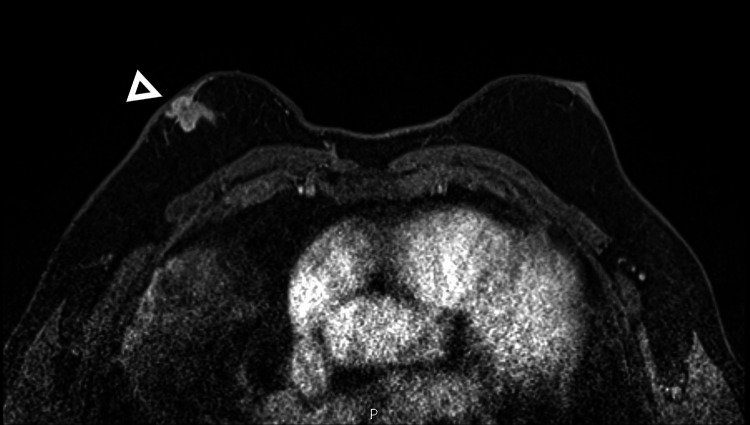
Axial T1 weighted image after IV gadolinium shows a heterogeneously enhancing mass in the right subareolar region with flattening right nipple-areolar complex.

The mass was nonmobile, painless, and retroareolar (Figures 1-2). There was no associated breast pain, nipple discharge, retraction, or skin changes, including discoloration.

Pathology

Pathology provided a histologic grade of 3, also known as poorly differentiated. The tissue sample tested positive for estrogen and progesterone receptors but negative for HER2. A biopsy of the axillary lymph nodes revealed metastatic illness. The sample was classified as clinical prognostic stage IIA cT1N1M0 according to the AJCC 8th edition. The patient was diagnosed with a germline pathogenic mutation of the BRCA2, CHEK2, and FANCC genes after genetic testing. BRCA2 is a high-risk breast cancer gene, while CHEK2 is a moderate-risk breast cancer gene.

General epidemiology

Male breast cancer is exceedingly uncommon, accounting for less than 1% of all malignancies in men and less than 1% of all breast cancer diagnoses worldwide [1,2]. Male breast cancer is very similar in terms of epidemiology to post-menopausal female breast cancer, with similar risk factors including age, family history, pathogenic mutations in the BRCA genes, external use of estrogen and testosterone, and obesity, among others [1,2]. The most important risk factor for the development of male breast cancer is a pathogenic mutation in the BRCA2 gene [3]. An estimated 5-10% of men with BRCA2 mutations develop breast cancer in their lifetime [4]. Additionally, the incidence of male breast cancer has increased steadily from 0.85 per 100,000 people in 1975 to 1.19 per 100,000 people in 2015, a 40% increase [5]. Most male patients present at a more advanced age and a more advanced stage when compared to females with breast cancer [2], likely due to the low incidence and lack of knowledge that men can develop breast cancer. Male breast cancers are more likely to occur in the subareolar region, whereas female breast cancers are more likely to occur in the upper outer quadrant [2].

## Discussion

Prognosis, treatment, or therapeutic options

Treatment of male breast cancer often involves an array of modalities, including surgical removal of the mass, chemotherapy, and radiotherapy. Removal of the mass can be achieved by either mastectomy or a breast-conserving intervention (central lumpectomy). A lumpectomy may be performed in older patients with a small tumor, but this treatment option is rarely preferred due in part to the mandatory radiotherapy that must follow surgery [2]. Patients who have positive lymph node biopsy results, tumors larger than 1 cm in size, and/or tumors that are hormone receptor negative are candidates for neoadjuvant and/or adjuvant chemotherapy [2]. Higher histologic grades indicate a higher rate of recurrence, and thus post-surgery radiation would be considered. The breast cancer mortality rate for males was recently found to be significantly worse than females across all stages [5]. The overall survival rate for men is estimated at 45.8%, while for women it is estimated at 60.4% [5]. Both clinical characteristics and undertreatment were found to be associated with a 63.3% excess mortality among male breast cancer patients [5].

## Conclusions

The breast cancer mortality rate for males was recently found to be significantly worse than females across all stages. This can be multifactorial, as previously stated. As there are no routine screening assessments for men available, this will continue to be a relevant problem. This case report highlights pertinent imaging features of male breast cancer.
